# Avirulence Genes in Cereal Powdery Mildews: The Gene-for-Gene Hypothesis 2.0

**DOI:** 10.3389/fpls.2016.00241

**Published:** 2016-03-01

**Authors:** Salim Bourras, Kaitlin E. McNally, Marion C. Müller, Thomas Wicker, Beat Keller

**Affiliations:** Institute of Plant and Microbial Biology, University of ZurichZurich, Switzerland

**Keywords:** wheat, powdery mildew, resistance gene, avirulence gene, barley

## Abstract

The gene-for-gene hypothesis states that for each gene controlling resistance in the host, there is a corresponding, specific gene controlling avirulence in the pathogen. Allelic series of the cereal mildew resistance genes *Pm3* and *Mla* provide an excellent system for genetic and molecular analysis of resistance specificity. Despite this opportunity for molecular research, avirulence genes in mildews remain underexplored. Earlier work in barley powdery mildew (*B.g. hordei*) has shown that the reaction to some *Mla* resistance alleles is controlled by multiple genes. Similarly, several genes are involved in the specific interaction of wheat mildew (*B.g. tritici*) with the *Pm3* allelic series. We found that two mildew genes control avirulence on *Pm3f*: one gene is involved in recognition by the resistance protein as demonstrated by functional studies in wheat and the heterologous host *Nicotiana benthamiana*. A second gene is a suppressor, and resistance is only observed in mildew genotypes combining the inactive suppressor and the recognized *Avr*. We propose that such suppressor/avirulence gene combinations provide the basis of specificity in mildews. Depending on the particular gene combinations in a mildew race, different genes will be genetically identified as the “avirulence” gene. Additionally, the observation of two LINE retrotransposon-encoded avirulence genes in *B.g. hordei* further suggests that the control of avirulence in mildew is more complex than a canonical gene-for-gene interaction. To fully understand the mildew–cereal interactions, more knowledge on avirulence determinants is needed and we propose ways how this can be achieved based on recent advances in the field.

## Avirulence Genes in Fungal Plant Pathogens

The identification of avirulence (*Avr*) genes in plant pathogenic fungi has been accelerating in recent years due to rapid advances in ‘omics’ technologies. At present, at least 35 *Avrs* have been cloned from filamentous fungi infecting a wide variety of agronomically important crops. Examples of *Avrs* fitting the gene-for-gene model are found in *Cladosporium fulvum* and *Leptosphaeria maculans*, where single *Avrs* are recognized by their cognate resistance (*R*) genes (Wulff et al., 2009; Hayward et al., 2012). However, in *Magnaporthe oryzae* and *Melampsora lini*, there are cases of single *Avrs* recognized by multiple *R* genes, as it was found for *Avr-Pik/km/kp* (Yoshida et al., 2009), *AvrL567*, and *AvrP123* (Barrett et al., 2009; Ravensdale et al., 2012). Another level of complexity has been described in *Fusarium oxysporum* f. sp. *lycopersici*, which encodes *Avr1* that is recognized by the tomato *R* gene *I-1*, but also acts as a suppressor of the recognition of *Avr2* and *Avr3* by *I-2* and *I-3*, respectively (Houterman et al., 2008).

Thus far, most cloned avirulence genes encode a typical effector protein (63–314 amino acids) with a predicted signal peptide for secretion, with *Ace1* (4034 amino acids) from *Magnaporthe grisea* being one of three exceptions (the other two are *AvrMla* genes, see below). *Ace1* encodes a hybrid polyketide synthase non-ribosomal peptide synthetase involved in the biosynthesis of the actual avirulence factor (Boehnert et al., 2004). In contrast to AVR proteins from Oomycetes that contain few or no cysteines, fungal AVRs are often cysteine-rich, which confers stability in the leaf apoplast. Most AVRs cloned from fungal pathogens forming specialized infection structures (e.g., *Blumeria graminis, M. lini, M. grisea*) appear to contain fewer cysteines than AVRs from fungal pathogens that invade exclusively with hyphae (for a summary, see Rouxel and Balesdent, 2010). Thus, differences in cysteine content might reflect the mechanistic differences between these two infection strategies.

## Avirulence in Cereal Mildews: Genetic Analysis in Segregating Populations

**FIGURE 1:**
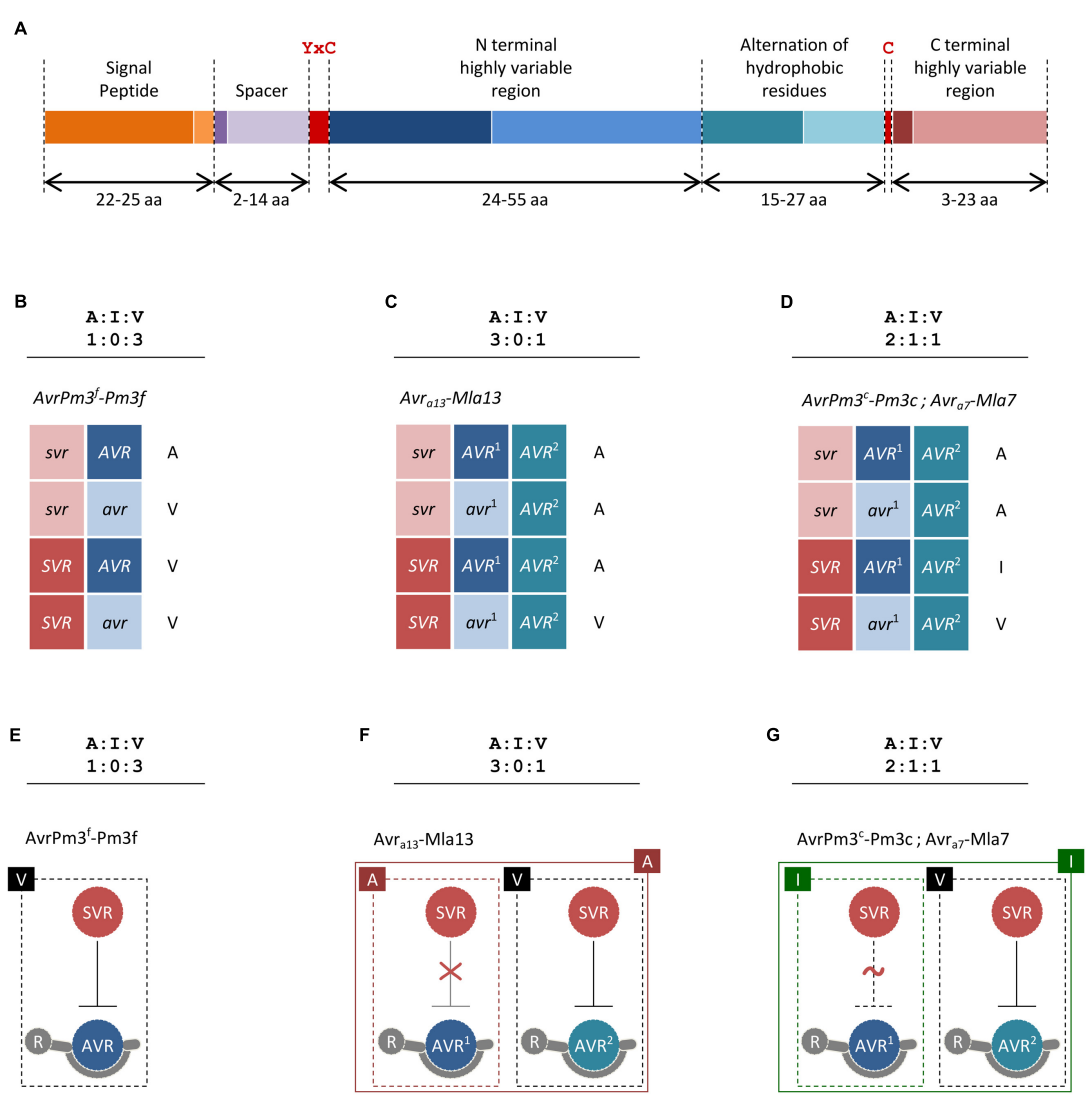
**Proposed models for *Avr*–*R*–*Svr* interactions in cereal powdery mildews. (A)** Structure of the AvrPm3^a2/f2^ effector family. Minimum and maximum sizes of each region as observed among family members are given in amino acids (aa) and differentiated with dark and light color shades, respectively. The YxC motif and the conserved cysteines are indicated in red. **(B–D)** Proposed *Avr-R-Svr* genetic models for the interpretation of genetic segregation ratios deviating from the canonical 1:1 single gene hypothesis. For readability, active and inactive alleles are distinguished with upper and lower case. Here, we have only considered examples of mildew *Avr–R* interactions where genetic segregation ratios and mapping data were consistent with at least two genetically independent loci being involved in avirulence. **(B)** Considering the *Avr-R-Svr* model and a genetic segregation ratio of 1:0:3, avirulence results from a combination of an inactive suppressor allele (*svr*) and an active avirulence allele (*AVR*). In the presence of the active *SVR* and the active *AVR*, the interaction result in virulence. A molecular model for this suppression scenario is depicted in **(E)**. **(C)** Considering the *Avr-R-Svr* model, a genetic segregation ratio of 3:0:1 can be explained by a model involving one suppressor locus and two loci for avirulence (*AVR*^*1*^ and *AVR*^*2*^). Importantly, the second locus for avirulence (*AVR*^*2*^) is not polymorphic in this cross, thus only the active allele (*AVR*^*2*^) is present. In this model, the active *SVR* is only effective in suppressing *AVR*^*2*^ but not *AVR*^*1*^. A molecular model for this suppression scenario is depicted in **(F)**. **(D)** Considering the *Avr-R-Svr* model, a genetic segregation ratio of 2:1:1 can be explained by a model involving one suppressor locus and two loci for avirulence (*AVR*^*1*^ and *AVR*^*2*^). Importantly, the second locus for avirulence (*AVR*^*2*^) is not polymorphic in this cross, thus only the active allele (*AVR*^*2*^) is present. In this model, the active *SVR* can fully suppress *AVR*^*2*^ but is only partially effective on *AVR*^*1*^, thus resulting in avirulence in the first case and intermediate virulence in the second. A molecular model for this suppression scenario is depicted in **(G)**. **(E–G)** Proposed suppression scenarios based on the genetic models described in **(B–D)** and resulting in three major phenotypic classes of avirulence ‘A,’ virulence ‘V,’ and intermediate virulence ‘I.’ Only active suppressor and avirulence proteins are shown. For simplicity, AVR–R interactions are represented as physical binding. Absence of suppression is depicted as a red “X” sign. Partial suppression is depicted as a dotted line and a red “∼” sign. The phenotype resulting from single and combined interactions is indicated.

The genetic basis of *Avr–R* interactions in cereal powdery mildews was investigated using classical genetic approaches in eleven crosses of *B.g. hordei* (see Skamnioti et al., 2008, for a summary), two of *B.g. tritici* (Bourras et al., 2015; Parlange et al., 2015) and at least in one cross of *B.g. secalis* and *B.g. tritici* hybrids (Tosa, 1994). In the resulting haploid F_1_ progeny, each genetic locus from the parental genotypes is expected to segregate in a 1:1 ratio, so that only one of the two parental alleles is present per locus and per individual. Deviations of *Avr* segregation from the classical 1:1 single gene model were frequently observed, indicating the involvement in avirulence of at least two or three genes (see **Table 1** for a summary and **Figures 1B–D**). Thus, in addition to the single gene control of avirulence that is commonly observed in plant pathogenic fungi, there are more complex genetic situations in cereal powdery mildews. Furthermore, classical genetic studies have often reported the emergence of additional phenotypic classes in the segregating progeny, where a quantitative variation in virulence contrasting with the parental phenotypes was observed (**Table 1**; Brown and Jessop, 1995; Bourras et al., 2015; Parlange et al., 2015). For instance, in the cross between the avirulent *B.g. tritici* isolate 96224, and the virulent isolate JIW2, 33 progeny showed consistent intermediate phenotype on wheat lines containing the mildew resistance gene *Pm3c* (**Table 1**). Similar examples were reported in segregating progeny growing on the mildew resistance genes *Pm3b*, *Pm3d*, and *Mla7* (**Table 1**). Thus, in cereal powdery mildews there is a quantitative component in race-specific *Avr–R* interactions that is reminiscent of a polygenic, quantitative trait.

**Table 1:** Non-canonical segregation of race-specific avirulence in the F_1_ progeny of wheat and barley powdery mildew crosses.

			Progeny phenotypes^3^			Genetic segregation^4^					
Host	Resistance^1^	Mildew crosses^2^	A	I	V	Ratio	Nbr. Loci	χ^2^	*p*-Value	Genet. Map.^5^	Source^6^
Wheat	Pm3b	96224 × 94202	108	17	33	5:1:2	3	2.319	0.313	2	Bourras et al., 2015
	Pm3c	96224 × JIW2	78	33	42	2:1:1	2	0.107	0.743	2	Parlange et al., 2015
	Pm3d	96224 × 94202	44	18	96	2:1:5	3	0.744	0.689	2	Bourras et al., 2015
	Pm3f	96224 × 94202	46	2	119	1:0:3	2	0.745	0.388	2	Bourras et al., 2015
Barley	Mla6	CC151 × DH14	30	–	10	3:0:1	2	0.000	1.000	1	Brown et al., 1996
	Mla7	CC107 × DH14	26	13	12	2:1:1	2	0.120	0.942	2	Brown and Jessop, 1995
	Mla13	CC52 × DH14	46	–	20	3:0:1	2	1.320	0.251	2	Caffier et al., 1996

*(1) Powdery mildew resistance genes in wheat (Pm) and barley (Ml) on which non-canonical segregation of avirulence was reported. (2) Wheat and barley powdery mildew crosses used for genetic studies. Crosses are indicated as parent_1 × parent_2. All crosses were done with isolates from the same forma specialis. (3) Progeny phenotypes are indicated as “A” for avirulent, “V” for virulent, and “I” for intermediate virulence that is referred to as “intermediate leaf coverage” in wheat powdery mildew or “intermediate infection types” in barley powdery mildew (for details on phenotype scoring please refer to the literature cited in the last column). (4) Genetic segregation is given as the theoretical ratio of A:I:V as reported in the literature cited in the last column. The number of loci/genes involved is indicted. Deviation of phenotype numbers from the theoretical ratios was tested with the χ^2^ test for goodness of fit. The degree of freedom was assigned as the “Number of phenotypic classes – 1.” The probability value for the χ^2^ test is indicated. P < 0.05 would indicate significant deviation from the theoretical ratios. (5) The number of loci genetically mapped among the ones inferred from the genetic ratios, as described in Skamnioti et al. (2008) and Bourras et al. (2015). (6) Literature from which phenotype segregation data was extracted.*

In addition to studies of inheritance, linkage between different *Avr* encoding loci was resolved for 15 *Avr–R* interactions in *B.g. hordei* and six in *B.g. tritici* (Skamnioti et al., 2008; Bourras et al., 2015). In both *formae speciales*, two genetically unlinked regions in the genome that commonly control several *Avr* specificities were reported (see **Table 1** for some examples). In *B.g. hordei* there is the *AVR_*a10*_* locus controlling the *AVR_*a10*_*, *AVR_*k1*_*, *AVR_*a22*_*, *AVR_*a9*_*, *AVR_*a13-1*_*, *AVR_g_* and possibly *AVR_a6_* and *AVR_a7-2_* specificities. There is also the *AVR_*a12*_* locus comprised of *AVR_*a6*_, AVR_*a12*_*, *AVR_*P17*_*, and *AVR_La_*. Similarly in *B.g. tritici* there is *locus_1* controlling the *AvrPm3^a^*, *AvrPm3^*b1*^*, *AvrPm3^*c1*^*, *AvrPm3^*d1*^*, *AvrPm3^*f1*^*, and *AvrPm3^e^* specificities, and *locus_3* for *AvrPm3^*b2*^*, *AvrPm3^*c2*^*, and *AvrPm3^d*2*^*. However, while in *B.g. hordei* the *AVR_*a10*_* and the *AVR_*a12*_* loci were described as clusters of linked but recombining *Avr* genes (Skamnioti et al., 2008), *locus_1* in *B.g. tritici* was characterized as encoding for a single *Avr* factor commonly involved in the interaction with six alleles of the *Pm3* resistance genes (Bourras et al., 2015; Parlange et al., 2015). Considering the *AvrPm3^a/f^-Pm3a/f* interaction involving *locus_1* and *locus_2* in *B.g. tritici*, it was found that specificity is only encoded by *locus_2*, while *locus_1* encodes a factor acting unspecifically on several *AvrPm3–Pm3* interactions. Assuming this genetic model stands true for *B.g. hordei*, it is possible that the *AVR_*a10*_* and *AVR_*a12*_* loci encode for general factors that are reminiscent of the *B.g. tritici locus_1*, while the actual avirulence proteins are encoded within specific loci outside of these clusters.

## Genes Encoding CSEPs in Cereal Mildews: The Primary Candidates for Avirulence Genes

Genes encoding so-called candidate secreted effector proteins (CSEPs; Spanu et al., 2010) have been suggested as major determinants of the mildew–host interaction and as good candidates for avirulence proteins. Refined analyses of the CSEP complement in the *Blumeria* genomes indicated there are close to 500 of such genes in *B.g. hordei* (Pedersen et al., 2012) and over 600 in *B.g. tritici* (Fabrizio Menardo, personal communication), hence much more than initially predicted (Spanu et al., 2010; Wicker et al., 2013). Pedersen et al. (2012) showed that many CSEPs are highly expressed in haustoria, and thus expected to be released from the haustorial membrane into the extrahaustorial matrix. How effector proteins subsequently enter the plant cell is still unclear, although data from site directed mutagenesis suggest that specific conserved protein motifs such as the HRxxH motif in the CSEP BEC1019 may mediate translocation of the effector through the plant membrane (Whigham et al., 2015).

Beyond the common features described above, CSEPs have very limited sequence similarity and only a few common protein motifs are found (e.g., the YxC motif, **Figure 1A**), suggesting they may target different proteins in the host and fulfill different functions (Pedersen et al., 2012; Wicker et al., 2013). One group of predicted CSEP proteins share structural similarities to ribonucleases (Pedersen et al., 2012), which is why they are suspected to mimic and compete with plant proteins involved in pathogen defense. Furthermore, several CSEPs have homology to enzymes that could be involved in processes of plant–pathogen interaction such as cell-wall remodeling or protein degradation (Pliego et al., 2013). Indeed, a *B.g. hordei* CSEP of the latter type (BEC1019) was recently shown to suppress plant cell death (Whigham et al., 2015). In summary, our understanding of the actual ‘effects’ of CSEPs is very limited, but we can expect that data from comparative analyses and functional assays will soon shed more light on this large class of fungal proteins.

## The Identification of *Avr_a*10*_* and *Avr_k*1*_*: A Novel Class of Avirulence Genes Encoded by Line Retrotransposons

The two avirulence genes *Avr_a*10*_* and *Avr_k*1*_* were isolated by map-based cloning from *B.g. hordei* by Ridout et al. (2006). Both encode unusual avirulence proteins as they lack signal peptides. *Avr_*k1*_* was functionally validated by bombardment assays that demonstrated induction of cell death in a *Mlk*-resistance gene dependent manner, as well as enhanced infection on susceptible varieties after transient expression. It was found that recognition of AVR_*a10*_ by MLA10 induced nuclear associations between the immune receptor and WRKY transcription factors (Shen et al., 2007). Furthermore, Nowara et al. (2010) described that silencing of *Avr_*a10*_* resulted in reduced fungal development in the absence but not in the presence of the cognate resistance gene *Mla10*.

In *B.g. hordei Avr_a*10*_* and *Avr_*k1*_* were found to map only 0.7 cM from *Avr_a22_* (Skamnioti et al., 2008), and the evolutionary origin of these *Avr* genes has been subject of intense debate. Recent genome-wide analysis and comparisons with LINE elements from animals and plants made clear that *Avr_a*10*_* and *Avr_*k1*_* are derived from non-LTR retrotransposons (also known as Long Interspersed Nuclear Elements or LINEs). These retro-elements usually consist of two open reading frames (ORFs), where ORF2 encodes a reverse transcriptase and RNaseH (RT/RH), while ORF1 is thought to encode a protein that mediates the transfer of retrovirus-like particles into the nucleus, at least in the human L1 element. *Avr_a10_* and *Avr_*k1*_* are both derivatives of ORF1, but their homology at the DNA and protein level is very limited, thus indicating that both genes arose independently from distantly related LINE families (Amselem et al., 2015). Since LINEs are among the most abundant types of transposable elements in the *Blumeria* genomes (Parlange et al., 2011), it is not surprising that more than a thousand *Avr_a*10*_* and *Avr_*k1*_* homologs were originally interpreted as a very large gene family that happened to co-evolve with the RT/RH domain of LINEs (Sacristán et al., 2009). The evolutionary origin of *Avr_a10_* and *Avr_*k1*_* from LINEs has the intriguing implication that the large amount of repetitive DNA found in powdery mildew genomes serves as raw material from which new proteins and regulatory regions can emerge (Amselem et al., 2015).

## The Identification of *SvrPm3^*a1/f1*^* and *AvrPm3^*a2/f2*^* in *B.g. tritici*: Two CSEPs Involved in *Pm3*-Mediated Avirulence

The identification of the first *Avr* gene in wheat powdery mildews was enabled by a combination of new genomic tools, next generation sequencing and high-throughput genotyping technologies (Bourras et al., 2015). Analysis of the two loci controlling the *AvrPm3^a/f^–Pm3^a/f^* interaction resulted in the cloning of *AvrPm3^*a2/f2*^*, a typical CSEP encoded within *locus_2* (**Figure 1A**). The avirulent allele is specifically recognized by *Pm3a* and *Pm3f*, whereas the virulent allele differing by two amino acid polymorphisms escapes recognition. Similarly, *SvrPm3^*a1/f1*^*, also a typical CSEP gene encoded within the general *locus_1* was cloned (Parlange et al., 2015). Most likely, this gene acts as a suppressor (*Svr)* of the *AvrPm3–Pm3* mediated avirulence (Bourras et al., 2015). Thus, in the *AvrPm3–Pm3* interaction model, a second layer of regulation is provided by a suppression mechanism. Here, in the presence of an active SVR suppressor, primary recognition of the avirulence effector by the cognate resistance protein is not sufficient to induce a resistance response. In this model, it is possible for the pathogen to maintain an unaltered and active AVR effector while still escaping *R* gene recognition which represents an evolutionary advantage on the long term.

It was shown that both *Avr* and *Svr* genes have the same kinetics of expression with a peak at 2 days after mildew inoculation (Bourras et al., 2015). This time point coincides with the formation of the haustorium, a fungal feeding structure involved in effector delivery, and a milestone for successful infection. It was also shown that *Avr* and *Svr* regulation is inherited in a parent-of-origin-specific manner in progeny from a cross between the *Pm3a/f* avirulent isolate 96224 and the *Pm3a/f* virulent isolate 94202 (Bourras et al., 2015). Considering these parental isolates and the critical time point of 2 days, it was found that the *Avr* gene is upregulated in the avirulent parent while the *Svr* suppressor is downregulated, and the exact opposite situation was found in the virulent parent. This situation can be explained by mechanisms such as epiallelic variation, mutations in *cis*-elements, alteration of trans-acting factors, or epigenetic modulation, all of which can affect effector protein accumulation without altering protein sequence (Bakkeren and Valent, 2014; Gijzen et al., 2014). Thus, in addition to sequence polymorphism distinguishing *Avr* and *avr* alleles, and the presence of an active (*Svr*) or inactive (*svr*) suppressor, there is a third layer of regulation of the *AvrPm3–Pm3* interaction at the gene expression level.

Based on these results, an extension of Flor’s gene-for-gene model that accounts for a suppressor locus was proposed as the *Avr-R-Svr* genetic model (**Figures 1B–D**). Here, resistance is mediated by an interaction involving an allele-specific avirulence effector (*Avr)*, a resistance gene allele (*R*), and an allele-unspecific pathogen-encoded suppressor of avirulence (*Svr*). At the molecular level, recognition and suppression of recognition are determined by sequence polymorphism as well as gene expression levels differentiating *Avr vs. avr* and *Svr vs. svr* alleles. Thus, resistance can only occur if the suppressor is inactive and the AVR protein is produced in sufficient amounts for *R* gene activation.

## How is Resistance Specificity to Allelic Series of Resistance Genes Controlled by Powdery Mildew?

The recent studies on the *Pm3a* and *Pm3f* resistance genes (Stirnweis et al., 2014) on the host side, as well as the identification of *AvrPm3^*a2/f2*^* on the pathogen side (Bourras et al., 2015) have given a first insight into the control of molecular specificity. Considering these two *Pm3* alleles, *Pm3a* is a stronger form of *Pm3f* as *Pm3a* recognizes all isolates which are recognized by *Pm3f*, plus some additional ones (Brunner et al., 2010). It was found that activation efficiency through the ARC2 domain controls the difference in the recognition spectrum of these two alleles, and there is no difference in recognition specificity (Stirnweis et al., 2014). Furthermore, there is strong evidence that specificity in the allelic interactions is based on the recognition of different *AvrPm3* or *AvrMla* genes. *AvrPm3^*a2/f2*^* is not recognized by any of the other *Pm3* alleles and genetic mapping (Bourras et al., 2015) and mutant analysis (Parlange et al., 2015) have revealed at least three additional genetic loci controlling recognition specificity toward *Pm3* alleles in *B.g. tritici*. In *B.g. hordei* also, the complex genetic ratios of avirulence inheritance indicate the involvement of many genes (**Table 1**). Thus, the corresponding avirulence genes seem to be quite different from each other, a surprising finding given that some allelic PM3 protein variants differ only by two amino acids in the LRR domain. Finally, resistance specificity also involves the pathogen encoded *SvrPm3^*a1/f1*^* suppressor of the *Pm3* resistance (**Figures 1B–G**) (Bourras et al., 2015). In *B.g. hordei*, the molecular basis of recognition specificity of the *Avr_*a10*_* and *Avr_*k1*_* alleles is not yet understood. As discussed below, there are several ways to further explore the function of these genes. In addition, it will be essential to characterize at the protein level the determinants of recognition, and also to isolate more *AvrMla* genes for the many members of the *Mla* allelic series (Seeholzer et al., 2010) to get further insight into this interaction.

Clearly, genetic studies in mildew are greatly simplified by haploid inheritance, but haploid genetics has the disadvantage that dominant and recessive traits cannot easily be distinguished. Thus, based on genetics only it is not possible to predict whether an “avirulence gene” actually encodes for an AVR that is recognized by the cognate resistance protein, or an inactive suppressor that is ineffective in inhibiting AVR-R recognition and/or resistance signaling. In addition to this first layer of complexity, gene expression possibly plays an important regulatory role in the determination of specificity (Bourras et al., 2015). Therefore, differences in gene expression levels between different *B.g. tritici* or *B.g. hordei* isolates might actually give important hints on functional avirulence and suppressor genes. In this context, we propose that comparative RNAseq studies in many isolates might be useful to detect such diagnostic expression differences.

## Next Steps in the Characterization of Cereal Powdery Mildew Avirulence

Despite the progress in the characterization of mildew avirulence genetics and genomics described above, there are large remaining gaps in our knowledge. Considering the identified components of avirulence in mildew, the specific interactions leading to *AvrPm3^*a2/f2*^* recognition/suppression need to be studied at the molecular and biochemical level. In addition, the role of the LINE encoded *B.g. hordei* avirulence genes *Avr_*a10*_* and *Avr_*k1*_* needs to be clarified. It is also possible that these two genes are not recognized by the resistance genes (in a situation similar to *SvrPm3^*a1/f1*^* in the case of *B.g. tritici*) but rather play a different, molecularly unknown role as activators or suppressors of avirulence. In this context, it will be important to isolate more *AvrPm3* and *AvrMla* genes to understand the molecular basis of specificity in cereal mildews.

So far, all known *Avr* loci in *Blumeria* were isolated by map-based cloning approaches which are solid but very time-consuming. The *B.g. tritici* and *B.g. hordei* reference genomes should be improved by new sequencing technologies such as PacBio (Eid et al., 2009) which would greatly support map-based cloning and pave the way for genome-wide association studies (GWAS), an attractive alternative for *Avr* identification. The decreased costs of next-generation sequencing allow us to sequence a large number of isolates which is the basis for GWAS. However, it remains to be seen if GWAS approaches will be capable of identifying mildew *Avrs*, where avirulence seems to result from complex genetic networks and epistatic interactions.

A largely unexplored area of research is the identification of specific mutants in avirulence genes. Such mutants should theoretically be easy to identify by mutagenizing avirulent races and selecting for growth on a barley or wheat genotype containing the cognate resistance gene. Two such mutants at new and still uncharacterized genetic loci have been identified for loss-of-recognition by *Pm3a* and *Pm3f*, with no impact on the other *Pm3* alleles (Parlange et al., 2015). Based on whole genome sequencing of pools from segregants of mapping populations, and considering the haploid nature of the mildew genome, it should be straightforward to identify the causative mutations and we suggest that larger screens for many different *Avr* mutants should be performed. Given that there are relatively few gene families in mildew (with the exception of rapidly diverging CSEP families, Wicker et al., 2013) genetic redundancy should be a minor problem for mutant identification. *AvrPm3^*a2/f2*^* belongs to a family of 36 members identified *in silico*. Twenty-three of these members, including the most homologous paralogs by protein sequence, PU_24 (61%) and PU_23 (59%), were functionally tested, but none of them was recognized by *Pm3a/f* (Bourras et al., 2015; our unpublished data).

Finally, there is a need for additional systems to study gene function. Particle bombardment has been a successful system to study *Avr* gene function, either directly or by host-induced gene silencing (Ridout et al., 2006; Nowara et al., 2010; Bourras et al., 2015), but it is time-consuming and needs practice and optimization in individual labs. In the case of the *AvrPm3^*a2/f2*^* avirulence gene, transient expression studies after infiltration of the heterologous species *N. benthamiana* have been successful (Bourras et al., 2015). However, it remains to be seen how broadly this system is suitable for mildew avirulence research. Therefore, alternative delivery approaches for functional studies, e.g., by bacterial vectors (Upadhyaya et al., 2014) should be developed for mildew. In addition, further work should be invested into the development of a highly reproducible mildew transformation system. This would then allow us to fully exploit the current rapid development in mildew avirulence biology and fully understand the interaction of this fascinating obligate biotroph and the co-evolution with its host.

In a more applied perspective, knowledge on the molecular interactions between *R* and *Avr* genes could be highly productive to develop pathogen-informed strategies for resistance improvement in the host. On the pathogen side, information on the allelic diversity of *Avrs* and *Svrs* in mildew populations can guide the spatio-temporal deployment of *R* genes in the field. On the molecular level, the identification of novel sources of resistance should be facilitated by the characterization of the genes and gene networks involved in resistance activation by AVR proteins *vs*. resistance suppression by the pathogen encoded suppressor. In particular, synthetic modification of wheat proteins co-opted by mildew suppressors might provide a strategy to achieve durable resistance.

## Author Contributions

All authors reviewed literature and contributed to writing the manuscript. SB and BK coordinated the manuscript.

## Conflict of Interest Statement

The authors declare that the research was conducted in the absence of any commercial or financial relationships that could be construed as a potential conflict of interest.
